# An Urban Center Experience Exploring Barriers to Adherence to Endoscopic Surveillance for Non-Dysplastic Barrett’s Esophagus

**DOI:** 10.7759/cureus.13030

**Published:** 2021-01-31

**Authors:** Mahmoud Isseh, Laurel Mueller, Hussna Abunafeesa, Zaid Imam, Dania Shakaroun, Mouhanna Abu Ghanimeh, Nazih Isseh, Joseph Miller, Syed-Mohammed Jafri, Adrienne Lenhart

**Affiliations:** 1 Internal Medicine, University of Michigan, Ann Arbor, USA; 2 Internal Medicine, Henry Ford Health System, Detroit, USA; 3 Gastroenterology and Hepatology, William Beaumont Hospital, Royal Oak, USA; 4 Gastroenterology and Hepatology, Henry Ford Health System, Detroit, USA; 5 Internal Medicine, The University of Tennessee Health Science Center, Memphis, USA; 6 Emergency Medicine, Henry Ford Health System, Detroit, USA; 7 Gastroenterology, University of California Los Angeles, Los Angeles, USA

**Keywords:** barrett’s esophagus, surveillance, barrett’s dysplasia

## Abstract

Background

Data regarding barriers to Barrett’s esophagus (BE) surveillance is limited. Studying an urban center population, we aimed to characterize non-dysplastic BE surveillance rates and identify health, racial, and socioeconomic disparities affecting surveillance.

Methods

Patients with biopsy-confirmed BE were retrospectively identified between January 2002 and December 2012. Non-dysplastic BE patients were analyzed for adherence to established surveillance guidelines. Demographic, racial, comorbidities, and socioeconomic variables were extracted. Annual gross income (AGI) was utilized as a marker of socioeconomic status (SES). Univariate and multivariate analyses compared adherent vs. non-adherent patients to surveillance guidelines.

Results

A total of 217 patients with non-dysplastic BE were analyzed. The majority were male (67.3%) and Caucasian (75.6%), with only 47.5% adherent with the first surveillance endoscopy. Patients with a high average AGI were more likely to be adherent with the initial surveillance endoscopy than those with low AGI (p=0.032). Initial compliance with first surveillance was associated with better surveillance at regular intervals (p=0.001). No significant differences in age, primary language, insurance type, marital status, or Charlson Comorbidity Index (CCI) between adherent and non-adherent patients were found.

Conclusions

Although overall adherence to guidelines was suboptimal, this study identifies important socioeconomic disparities in the endoscopic surveillance for non-dysplastic BE. Identifying and understanding the barriers to care among these lower socioeconomic groups may ultimately lead to improved screening compliance and early BE detection.

## Introduction

Barrett’s esophagus (BE) is a significant precursor to the development of esophageal adenocarcinoma (EAC), which occurs in approximately 0.2%-7% of patients with BE each year, depending on the degree of dysplasia [1,2]⁠. Given the increased risk of malignancy, guidelines recommend endoscopic surveillance of BE to help increase the likelihood of early cancer detection and improve overall survival [2]⁠. However, several retrospective and survey studies have shown poor adherence to established guidelines, with only 26%-77% of endoscopists demonstrating compliance [3-10]⁠. BE has also been shown to have a higher prevalence among older, Caucasian, men, with lower rates among Hispanic, Asian, and African American patients [9,11-13]⁠.

Prior studies have suggested that lower socioeconomic status (SES) is associated with lower rates of colorectal, breast, and cervical cancer screening [14,15]⁠. Additionally, higher EAC stage at the time of diagnosis has been reported in lower SES populations [16]⁠. Less awareness, lack of knowledge and support, negative beliefs about malignancy screening, and ideas about cancer fatality are some of the psychosocial aspects studied impacting attitudes of individuals of lower SES towards cancer screening [15]⁠. Adherence with surveillance guidelines for BE is poor in clinical practice, and disparities in the demographic distribution of BE have been identified. However, data is limited pertaining to existing barriers to surveillance. A recent study by Dalal et al. assessed adherence to endoscopic surveillance guidelines in nondysplastic BE patients in a Veterans Affairs (VA)-based population, and found that adherent cases were more likely to be older in age with a trend towards having long segment BE on initial endoscopy [10]⁠. Using an urban center population, we aimed to characterize non-dysplastic BE surveillance rates and identify health, racial, and socioeconomic disparities potentially affecting surveillance.

## Materials and methods

We performed a retrospective chart review of patients with biopsy-confirmed BE, identified from January 2002 through December 2011 using the International Classification of Diseases (ICD) 9 and ICD 10 codes. This study was conducted at a high-volume, urban, tertiary care hospital, only after approval from the Institutional Review Board. Patients included in this study were 18 years or older and were identified to have either non-dysplastic, low-grade dysplastic, or high-grade dysplastic BE on index upper endoscopy. Patients over the age of 75 years, patients with presumed BE but without biopsy confirmation, patients with indefinite biopsy results, or patients with EAC on initial endoscopy were excluded. Additionally, patients diagnosed with BE at an outside institution were excluded given the inability to confirm endoscopy and histopathological reports in the electronic medical record. Non-dysplastic BE patients were then further analyzed to determine adherence with established guidelines.

The primary outcome of our study was to determine the rate of adherence to surveillance guidelines in patients with non-dysplastic BE. A patient’s index endoscopy was defined as the first documented esophagogastroduodenoscopy (EGD) in our hospital system with esophageal biopsies that confirmed non-dysplastic BE. Adherence to surveillance guidelines was defined as a follow-up endoscopy after three years if the initial endoscopy was performed between 2002-2010, or after 3-5 years for index endoscopies performed during or after 2011. These different BE surveillance intervals were chosen based upon a change in the American College of Gastroenterology’s (ACG) and American Gastroenterological Association’s (AGA) established guidelines during this time frame. Both the 2002 and 2008 ACG practice guidelines for BE recommend a follow-up endoscopy after three years for patients with non-dysplastic BE [17,18]⁠. However, the AGA medical position statement on the management of BE in 2011 recommends a follow-up endoscopy after three to five years for patients with non-dysplastic BE [19]⁠. The follow-up interval of 2002 through 2011 was selected in order to analyze a decade of follow-up and surveillance for BE. A window period of six months on either side of the three year or three to five year follow-up period was allowed to count towards adherence. In cases in which a confirmatory endoscopy was performed within 2.5 years of the index EGD, this endoscopy did not count towards surveillance.

Secondary outcomes of our study were to determine if initially, adherent patients were more likely to undergo continued surveillance at regular or irregular intervals, as well as to determine any racial, social, or financial variables that could predict adherence to BE surveillance guidelines. The duration of additional surveillance was characterized from the first surveillance endoscopy through December of 2017. Regular surveillance was defined as continued adherence to guidelines based on the results of each additional esophageal biopsy [17-19]⁠. Irregular surveillance was defined as having either no additional surveillance endoscopies or endoscopies at periods other than those recommended by guidelines. Patients who died during the follow-up period or had their first surveillance endoscopy performed during or after 2012 were excluded from this part of the analysis.

Patient variables, including age, gender, race, body mass index (BMI), primary language, marital status, insurance type, annual gross income (AGI), and the Charlson Comorbidity Index (CCI) were collected and analyzed. AGI was determined based on the average household income of patients residing in specific zip codes and ranged from less than 40K, 40-60K, 60-80K, 80-100K, to over 100K United States Dollars (USD). An online website providing this data based on United States census bureau data was utilized to extract average household incomes used to define AGI [20]⁠. These variables were then assessed to determine if any disparities existed between those non-dysplastic BE patients who were adherent vs. non-adherent to surveillance guidelines. Only adherence to the initial surveillance endoscopy was analyzed in this portion of the study. Health Alliance Plan (HAP) is a non-profit organization in Detroit, Michigan providing multiple health maintenance organization (HMO) and preferred provider organization (PPO) coverage plans to patients [21]⁠.

Statistical analysis

Comparison testing was performed using the Fisher exact test and Chi square test for categorical variables, two-sample t-tests for normally distributed numeric variables, and Wilcoxon rank sum tests for non-normally distributed numeric variables. A multivariate logarithmic regression model included all variables predicting adherence with two-sided p-values <0.25. Statistical significance was defined as a two-sided p-value of <0.05 for all tests. Analyses were performed using Statistical Package for the Social Sciences; version 9.4 (SPSS Inc., Chicago, IL).

## Results

Demographics

We reviewed patients who carried a diagnosis of BE. Patients over the age of 75 years, patients without esophageal biopsies confirming BE, patients diagnosed with BE at an outside institution, patients with indefinite esophageal biopsy results, and patients with EAC on initial endoscopy were excluded from the analysis. A total of 224 patients met the inclusion criteria. Of these subjects, five patients had BE with low-grade dysplasia, two patients had BE with high-grade dysplasia and 217 patients had non-dysplastic BE. Since the management of individuals with low-grade and high-grade dysplasia is different in terms of surveillance duration and endoscopic therapy, these were excluded from the analyzed cohort. No sub-analysis was performed given the small number of patients with low-grade and high-grade dysplasia rendering analysis of low utility. Hence, 217 patients with non-dysplastic BE were in the final analysis. The mean age of individuals at the time of index endoscopy was 63.98 ± 10.13 years, and the majority were male (67.28%) and Caucasian (75.58%). In addition, the majority of patients in this study were married (63.59%), spoke English as their primary language (94.93%), had HAP for insurance coverage (40.09%), and had short-segment BE on esophageal biopsy (70.97%). Additional characteristics of the study population are described in Table 1.

**Table 1:** Patient demographics K, thousands; SD, standard deviation.

Variable	Patient No. (%)
Mean Age ± SD, years	63.98±10.13
Mean Charlson Comorbidity Index ± SD	4.38±2.48
Gender	
Male Sex	146 (67.28%)
Race	
Caucasian	164 (75.58%)
African American	37 (17.05%)
Asian	5 (2.30%)
Unknown	11 (5.07%)
Primary Language	
English	206 (94.93%)
Other	4 (1.84%)
Unknown	7 (2.83%)
Type of Health Insurance	
Health Alliance Plan	87 (40.09%)
Private, Non-Health Alliance Plan	49 (22.58%)
Medicare	69 (31.80%)
Medicaid	5 (2.30%)
None	4 (1.84%)
Marital Status	
Married	138 (63.59%)
Single	77 (35.48%)
Comorbidities	
Hypertension	151 (69.59%)
Diabetes Mellitus	57 (26.27%)
Obesity	71 (32.72%)
Liver Disease	15 (0.31%)
Chronic Obstructive Pulmonary Disease	31 (14.29%)
Congestive Heart Failure	22 (10.14%)
Coronary Artery Disease	23 (10.60%)
Chronic Kidney Disease	35 (16.13%)
Malignancy	64 (29.49%)
Annual Gross Income	
<40K	56 (25.81%)
40-60K	75 (34.56%)
60-80K	41 (18.89%)
80-100K	13 (5.99%)
>100K	24 (11.06%)

Adherence to surveillance guidelines

Of the 217 patients with non-dysplastic BE, only 103 (47.47%) were adherent with the first recommended surveillance endoscopy, whereas 114 (52.53%) were non-adherent with surveillance guidelines. Of the 103 patients who were adherent with the initial surveillance endoscopy, 35 patients had continued surveillance endoscopies at regular intervals, 28 patients proceeded with irregular surveillance, and 17 patients had no additional surveillance endoscopies. Four patients were excluded from this portion of the analysis secondary to death during the follow-up period confirmed through the medical record, and 19 patients were excluded secondary to undergoing their first surveillance endoscopy during or after 2012. Of the 114 patients with non-dysplastic BE who were non-adherent with the first surveillance endoscopy, nine patients had continued regular surveillance endoscopies, 28 patients proceeded with irregular surveillance, and 56 patients had no additional surveillance endoscopies. Ten patients were excluded from this portion of the analysis secondary to death during the follow-up period, and 11 patients were excluded secondary to undergoing their first surveillance endoscopy during or after 2012. Patients who were adherent to their first surveillance endoscopy were more likely to undergo additional surveillance endoscopies at regular intervals than were patients who were non-adherent to their first surveillance endoscopy (p=0.001).

Variables that may predict adherence to surveillance guidelines

In univariate analysis, patient age, gender, marital status, primary language, annual office visit with a primary care provider, obesity, tobacco use, and alcohol use had no significant impact on predicting adherence to the first surveillance endoscopy in patients with non-dysplastic BE. In addition, the CCI and the individual patient comorbidities of hypertension, diabetes mellitus, chronic obstructive pulmonary disease, congestive heart failure, coronary artery disease, malignancy, and chronic kidney disease (CKD) also did not help to predict adherence to surveillance guidelines. While not statistically significant, we did observe a trend towards better adherence among Caucasian patients compared to non-Caucasian patients (51.83% of Caucasian patients were adherent compared to 35.71% of non-Caucasian patients; p=0.062) (Table 2).

**Table 2:** Univariate analyses comparing variables between adherent and non-adherent groups to surveillance guidelines N, Number; HAP, Health Alliance Plan; K, Thousands; COPD, chronic obstructive pulmonary disease; PCP, primary care physician; CCI, Charlson Comorbidity Index; SD, standard deviation.

Variable	Non-Adherent to Surveillance	Adherent to Surveillance	P-value
	N=114	N=103	
Mean Age, years ± SD	64.0 ± 10.9	63.9 ± 9.4	0.961
Mean CCI±SD	4.7 ± 2.7	4.2 ± 2.2	0.208
Male Sex	76 (66.7%)	70 (68.0%)	0.839
Race			0.062
Caucasian	79 (74.5%)	85 (85.0%)	
Other	27 (25.5%)	15 (15.0%)	
English primary language	110 (99.1%)	96 (97.0%)	0.345
Married	69 (61.1%)	69 (67.6%)	0.315
Annual PCP visit	82 (72.6%)	78 (75.7%)	0.596
Tobacco Use	56 (49.1%)	61 (59.8%)	0.116
Alcohol Use	57 (50.0%)	52 (51.0%)	0.886
Hypertension	81 (71.1%)	70 (68.0%)	0.621
Diabetes Mellitus	31 (27.2%)	26 (25.2%)	0.744
Obesity	36 (31.6%)	35 (34.7%)	0.632
COPD	16 (14.0%)	15 (14.6%)	0.912
Congestive Heart Failure	13 (11.4%)	9 (8.7%)	0.516
Coronary Artery Disease	10 (8.8%)	13 (12.6%)	0.358
Liver Disease	10 (8.8%)	5 (4.9%)	0.256
Malignancy (solid tumor)	28 (24.6%)	31 (30.1%)	0.360
Chronic Kidney Disease	23 (20.2%)	12 (11.7%)	0.088
Health Insurance Type			0.369
Health alliance plan	41 (36.3%)	46 (45.5%)	
Private, Non-HAP	31 (27.4%)	18 (17.8%)	
Medicare	35 (31.0%)	34 (33.7%)	
Medicaid	3 (2.7%)	2 (2.0%)	
Non-insured	3 (2.7%)	1 (1.0%)	
Annual Gross Income			0.032*
<40 K	36 (32.1%)	20 (20.6%)	
40-60 K	40 (35.7%)	35 (36.1%)	
60-80 K	21 (18.8%)	20 (20.6%)	
80-100 K	5 (4.5%)	8 (8.2%)	
>100 K	10 (8.9%)	14 (14.4%)	

The type of health insurance that patients’ possessed did not have any significant impact on predicting adherence to surveillance guidelines in patients with non-dysplastic BE. However, patients with a higher average AGI were more likely to be adherent with the initial surveillance endoscopy compared to patients with a lower average AGI (35.71% of patients with an AGI <40K were adherent, 46.67% of patients with an AGI of 40-60K were adherent, 48.78% of patients with an AGI of 60-80K were adherent, 61.54% of patients with an AGI of 80-100K were adherent, and 58.33% of patients with an AGI >100K were adherent; p=0.032) (Table 2).

Caucasian race, tobacco use, moderate to severe CKD, AGI, and CCI score were the variables included in multivariate analysis. A non-significant trend toward better adherence was noted among Caucasian patients (p = 0.097) and patients with higher AGI (p = 0.086). These results are summarized in Table 3.

**Table 3:** Multivariable logistic regression results using patient characteristics to predict adherence Only variables with p-values less than 0.25 from Table 2 were included in this analysis. CCI: Charlson Comorbidity Index; CKD: chronic kidney disease.

Variable	P-value	Odds Ratio	95% Confidence Interval	
Caucasian Race	0.097	1.911	0.890	4.105
Tobacco Use	0.288	1.377	0.763	2.486
Moderate to Severe CKD	0.620	0.796	0.324	1.958
Annual Gross Income	0.086	1.229	0.972	1.555
CCI Score	0.388	0.945	0.831	1.074

Of the 35 patients who continued surveillance endoscopies at regular intervals, 65.7% were male, 80% were Caucasian, their mean age was 60.5 ± 10.2 years, 68.6% had short segment BE, the mean number of additional endoscopies performed were 2 ± 0.13, none of these 35 patients developed EAC.

## Discussion

In this busy tertiary care hospital-based study of 217 patients with biopsy-proven non-dysplastic BE, we found that less than half of the cases (47.47%) were adherent with guidelines. Our results are similar to Dalal et al.'s rate of 30% guideline adherence for non-dysplastic BE [10]⁠. It is important to note that our guideline adherence rates which are based on a retrospective review of cases with biopsy-proven non-dysplastic BE are significantly lower than the rates claimed by gastroenterologists in surveys conducted both in the US and Europe (86% and 76%, respectively) [22,23]⁠.

Our study showed that 43.68% of the patients who got the initial surveillance endoscopy either continued with irregular surveillance or failed to get any additional surveillance endoscopies (28 cases and 17 cases, respectively-out of the 103 cases with initial endoscopy). It is unclear whether physicians have a role in this trend in our healthcare system, as many gastroenterologists who have poor surveillance rates claim lack of efficacy of surveillance [8]⁠. This is especially true with non-dysplastic BE which has a very low annual incidence of EAC (0.33%), and even lower with short-segment BE (0.19%) [2] which represents 70.97% of all our biopsy-proven BE⁠.

Although many variables including type of insurance, annual office visit with a primary care provider, and patients’ comorbidities failed to predict adherence to surveillance guidelines, it is noted that Caucasian patients were more likely to adhere to a first surveillance endoscopy than non-Caucasian patients (51.83% and 35.71% retrospectively; p=0.062). It is important to note that over the last few decades, several studies have reported the rarity of BE and EAC diagnosis in the African American population which encompasses most of our non-Caucasian patients [12]⁠. This was indeed true in our study where 75.58% of the patients with non-dysplastic BE on biopsy were Caucasians and only 17.05% were African Americans (Table 1). Despite a lower rate of BE and dysplastic BE in African Americans compared to non-Hispanic whites [12]⁠, the variation in rates of adherence to guidelines can be multifactorial. Mistrust of the medical community, limited access to care, lack of social support, and poor health literacy can be contributors to the lower rates of surveillance endoscopies in the non-Caucasians and mainly the African American population.

Our study corroborated the negative impact of lower SES to adherence to screening programs, similar to other studies evaluating colorectal and breast cancer [24,25]⁠. Establishing national screening programs do not ameliorate the effects of these disparities on earlier diagnosis of malignancy [26]⁠. Multifactorial mechanisms including psychosocial factors impacting access and adherence of individuals from disadvantaged populations translate into worse patient outcomes [15,16]. Identifying these disparities and more in-depth analysis of the etiological factors behind them is the first step in improving the adherence of disadvantaged populations to screening programs.

The study’s strengths include its ability to evaluate the real-life application of screening guidelines at an urban medical center and a practical tertiary care center, as well as its novelty; to our knowledge, there are no similar studies that evaluated the association between SES and BE surveillance adherence.

Our study has several limitations. These include the retrospective and single-center nature of the study, the inability to evaluate EGDs done at outside hospitals potentially resulting in missing data, and potential for data misclassification. Non-adherence does not necessarily translate into under surveillance, as EGDs performed and not captured by the study methodology might suggest over surveillance by some providers that is not guideline supported. Additionally, the use of zip codes to assess AGI is inferior to direct questioning of involved study patients about their annual income in a prospective fashion.

Patients with a high average AGI were more likely to be adherent with the initial surveillance endoscopy compared to patients with a low average AGI (p=0.032). This trend is expected, as the cost associated with endoscopy varies between $349 and $1120 [27,28]⁠.

## Conclusions

In conclusion, higher gross income reflective of higher SES appears to be an important predictor of surveillance adherence. Although the trend approached but did not meet statistical significance on multivariate analysis, this is likely in light of the moderate sample size. Future directions include evaluating similar trends in different urban centers around the United States, combining data from multiple centers, and evaluating the impact of socioeconomic disparities in non-Caucasian populations.
